# Grain Transcriptome Dynamics Induced by Heat in Commercial and Traditional Bread Wheat Genotypes

**DOI:** 10.3389/fpls.2022.842599

**Published:** 2022-06-17

**Authors:** Diana Tomás, Wanda Viegas, Manuela Silva

**Affiliations:** LEAF – Linking Landscape, Environment, Agriculture and Food, TERRA – Laboratory for Sustainable Land Use and Ecosystem Services, Instituto Superior de Agronomia, Universidade de Lisboa, Lisbon, Portugal

**Keywords:** bread wheat, commercial varieties, landraces, heatwave, grain transcriptome, RNA sequencing

## Abstract

High temperature (HT) events have negative impact on wheat grains yield and quality. Transcriptome profiles of wheat developing grains of commercial genotypes (Antequera and Bancal) and landraces (Ardito and Magueija) submitted to heatwave-like treatments during grain filling were evaluated. Landraces showed significantly more differentially expressed genes (DEGs) and presented more similar responses than commercial genotypes. DEGs were more associated with transcription and RNA and protein synthesis in Antequera and with metabolism alterations in Bancal and landraces. Landraces upregulated genes encoding proteins already described as HT responsive, like heat shock proteins and cupins. Apart from the genes encoding HSP, two other genes were upregulated in all genotypes, one encoding for Adenylate kinase, essential for the cellular homeostasis, and the other for ferritin, recently related with increased tolerance to several abiotic stress in Arabidopsis. Moreover, a NAC transcription factor involved in plant development, known to be a negative regulator of starch synthesis and grain yield, was found to be upregulated in both commercial varieties and downregulated in Magueija landrace. The detected diversity of molecular processes involved in heat response of commercial and traditional genotypes contribute to understand the importance of genetic diversity and relevant pathways to cope with these extreme events.

## Introduction

Wheat is the second most produced and consumed cereal worldwide on a daily basis (FAO^©^, 2019) and hexaploid bread wheat (*Triticum aestivum* L, 2n = 42) represents 90–95% of this production. However, the current growth rate of wheat production is not sufficient to cover the predicted global demand in 2050. Specifically in European countries, the stagnation of wheat yield increase is related with the progressive global warming (Brisson et al., 2010; Ray et al., 2013; Gaupp et al., 2019). The increase of mean temperature during wheat development affects grain yield and quality, due to reduction in lifecycle, pollen abortion, kernel shrinkage, and decrease in seed reserves (Asseng et al., 2014; Nuttall et al., 2018; Wang et al., 2019). The required optimum temperature for wheat anthesis and grain filling ranges from 12 to 22°C (Tewolde et al., 2006) and the overall acceleration of grain development observed under high temperature regimes is associated with the speed up of transcriptomic events (Altenbach and Kothari, 2004; Wan et al., 2008).

Transcription modulation of genes encoding heat shock proteins (HSPs) is the most studied molecular response under heat stress (Wahid et al., 2007). A recent study identified and characterized 753 HSP genes expressed in bread wheat, revealing the developmental stage and stress situation at which they are responsive (Kumar et al., 2020). HSPs transcripts were also differentially detected after 1 h and 1 d at 40°C using Wheat Genome Array profiles in seedlings of two genotypes with contrasting thermotolerances (Qin et al., 2008). The same work also detected transcription factors and genes involved in phytohormone biosynthesis/signaling, calcium and sugar signal pathways, RNA metabolism, ribosomal proteins, primary and secondary metabolisms synthesis, and biotic and abiotic stress responses. Chauhan et al. (2011) identified heat responsive genes, after 2 h of heat stress treatments (34 and 40°C), implicated in metabolites and protein synthesis in seedling shoot, flower tissues and developing grain through subtractive hybridization.

Whole transcriptome sequencing of wheat seedlings reported similar transcripts profiles after heat, drought and their combination treatments of 1 and 6 h (Liu et al., 2015). The main biological groups associated with upregulated genes were stress response, hormone stimulus response and nutrient metabolic processes, while downregulated genes were mainly enriched in photosynthesis and nutrient biosynthesis pathway. A more recent study used RNA Sequencing data obtained from developing grains of genotypes with distinct thermotolerances that underwent post anthesis heat stress for 3 days, identified different clusters of genes unique to tolerant and susceptible genotypes (Rangan et al., 2019). This work also refers that most genes uniquely expressed in tolerant genotype during heat stress are detected in both early and late grain filling reinforcing their role in heat stress response. Other work from Kino et al. (2020) compared RNA Sequencing data obtained from whole grains after post anthesis high temperature treatment (35°C during 2–12 days) against existing sequence data from individual pericarp and endosperm tissue. A significant down-regulation of pericarp genes with a known role in regulation of cell wall expansion was observed. For that reason, the authors suggested that heat treatment induces reduced expansion capability of the pericarp, which may result in a physical constraint of endosperm growth.

Several studies have shown increasing genetic erosion caused by the replacement of diverse old landraces by comparatively few and homozygous modern cultivars (Gregová et al., 1999; Caballero et al., 2001; Srinivasan et al., 2003). Landraces are dynamic populations of cultivated species lacking formal crop improvement, locally adapted and often genetically diverse [reviewed in Villa et al. (2005)]. Thus, landraces provide notable successes in crop improvement [reviewed in Dwivedi et al. (2016)] as sources of nutritional and technological quality traits and marginal environment tolerance [reviewed in Newton et al. (2010)]. They are considered extremely valuable agrobiodiversity pools in changing environmental conditions (Trethowan and Mujeeb-Kazi, 2008; Lopes et al., 2015) that may constitute a key resource facing extreme heat events like heatwaves. Heatwaves are defined by World Meteorological Organization [WMO] (2015) as five or more consecutive days of heat in which the daily maximum temperature is at least 5°C higher than the average maximum temperature. These adverse environmental events are foreseen to be increasingly frequent (Cardoso et al., 2019). The main goal of this work was to evaluate whole transcriptomic alterations induced by heatwave-like treatment during grain filling. This study was comparatively performed in two commercial varieties and two Portuguese landraces, chosen based on previous evaluations of high temperature (HT) responses regarding yield and grain composition (Tomás et al., 2020a,b,c).

## Materials and Methods

### Plant Material and High Temperature Treatment

The genotypes studied in this work comprehend two bread wheat (*Triticum aestivum* L., 2n = 6× = 42, AABBDD) commercial varieties recommended to be used in Portugal (ANPOC et al., 2014), Antequera and Bancal, and two old Portuguese landraces from Vasconcellos collection, established in the 30s of the last century (Vasconcellos, 1933), Ardito and Magueija. Seeds of commercial varieties were gently supplied by ANSEME (Portugal) and seeds of traditional landraces by EAN Germplasm Bank (Oeiras, Portugal, PRT005). Twenty seeds from each genotype obtained after 2 years of controlled propagation were germinated and grown in controlled conditions–8 h of dark at 20°C and a 16 h light period divided in 6 h increasing to 25°C, 4 h at 25°C, and 6 h decreasing to 20°C. Three-week old plants were transferred individually to seven liters soil pots and maintained in greenhouse conditions.

When the first anther was observed in the first spike (anthesis), plants were transferred to growth chambers with the previously described control conditions. Ten days after anthesis (daa) subsets of ten plants (biological replicates) of each genotype were submitted to two different growth conditions for 7 days: control conditions above described or high temperature (HT) regime with a daily plateau of 40°C maximum temperature (Supplementary Figure 1). Immediately after the period of 4 h at maximum temperature in the last day of the treatment, two immature grains from the middle of each first spike of each plant were collected (17 daa) and stored at –80°C for posterior RNA extraction.

### RNA Extraction, Library Preparation, and Sequencing

Total RNA was individually extracted from control and heat-treated immature grains using the Spectrum™ Plant Total RNA Kit (Sigma-Aldrich, Inc., Spain) and following manufacturer’s instructions. For the RNA sequencing, three biological replicates were analyzed per condition and genotype, each composed of a pool of three immature grains (total 100 ng of RNA). Both library preparation and sequencing were performed and optimized by the Genomics Unit of the Instituto Gulbenkian Ciência, Oeiras. mRNA-libraries were prepared using the SMART-seq2 protocol adapted from Macaulay et al. (2016) Illumina^®^ libraries performed used the Nextera protocol adapted from Baym et al. (2015). The libraries quantification and quality verification were done using the Agilent Fragment Analyzer in combination with HS NGS Kit (Agilent Technologies, Santa Clara, California). Libraries were sequenced in the NextSeq500 Illumina^®^ Sequencer using 75 SE high throughput kit (Illumina, San Diego, California) and 937302653 reads were obtained from the 24 samples.

### RNA Sequencing Data Processing and Differential Gene Expression Analysis

Bioinformatic analysis from quality assessment to differential expression analysis were performed by BioData.pt. Quality control was evaluated on raw reads using FastQC (Andrews et al., 2010). Raw reads were then trimmed using fastp (Chen S. et al., 2018) to the longest continuous segment of Phred-quality (threshold of 30 or above) in order to improve overall base quality, and remove the Illumina^®^ Smart-Seq2 adaptors from sequencing. A new quality control with FastQC was performed. The trimmed reads were mapped to *Triticum aestivum* genome^1^ using hisat2 with default parameters (Kim et al., 2015). Quality control of the mapping procedure was accessed with Qualimap (Okonechnikov et al., 2016).

Read assignment to genomic features and gene expression quantification were made using featureCounts (Liao et al., 2014). Differential gene expression was tested using DESeq2 (Love et al., 2014) between transcript sets of control and HT treated samples. Manual search of gene ID and encoding products was made in Ensemble Plants BioMart (Kinsella et al., 2011).

R software (R Core Team, 2018) was used to integrate all the analysis and obtain multi-dimensional scaling analysis (MDS) plot (to show the general relationship between the samples) and hierarchical clustering of samples for all varieties and conditions represented as an heatmap and Venn diagrams (showing the relationships between the differentially expressed genes lists of all varieties and conditions).

### Gene Ontology Enrichment Analysis

Gene enrichment (GO) analysis was done in AgriGOv2^2^ web-based tool (Tian et al., 2017). AgriGO SEA parameter settings were as follows: Fisher test, with Bonferroni multi-test adjustment method, 0.05 significance level, five minimum mapping entries, and complete gene ontology. The GO database^3^ was used to analyze GO terms enrichment of DEGs, and the Kyoto Encyclopedia of Genes and Genomes (KEGG) database^4^ was used to identify the enriched metabolic pathways, as well as the enzymes involved.

## Results and Discussion

In this work, plants of four wheat genotypes, Antequera, Ardito, Bancal, and Magueija were submitted to HT treatment simulating a heatwave, for 1 week during grain filling stage. Transcriptome profiles of immature grains collected immediately after treatment period (17 days after anthesis) from control and treated plants were analyzed.

### Traditional Genotypes Presented a More Similar High Temperature Response Than Commercial Varieties

**FIGURE 1:**
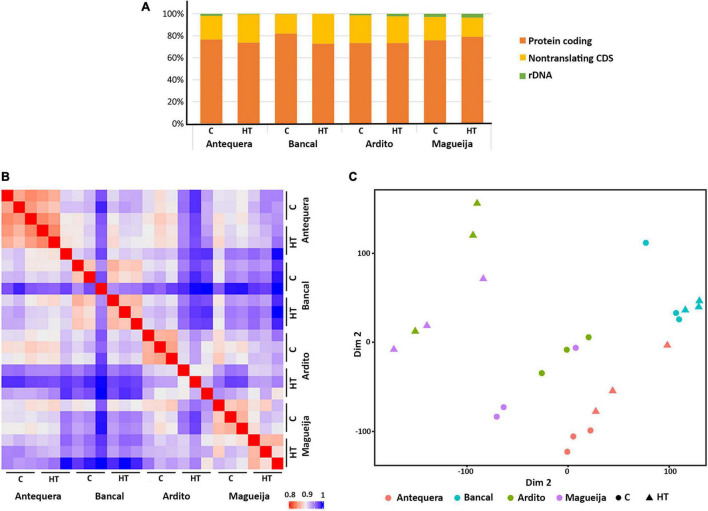
Reads assignments by gene type and relationship between samples. **(A)** Read assignments and relative abundance of reads per type of gene. **(B)** Hierarchical clustering of sample-to-sample correlations based on Pearson correlations (right). **(C)** Multi-dimensional scaling (MDS) plot showing similarity between all samples. The comparisons were made between control (C) and high temperature (HT) reads sets of commercial varieties Antequera and Bancal and landraces Ardito and Magueija.

The reference genome used to map transcripts was the IWGSC RefSeq v1.0 assembly (the first version of the reference sequence obtained from the bread wheat variety Chinese Spring). Overall, about 90% of the transcripts were mapped against the reference genome, and from these 37% mapped to multiple sites and the other 53% mapped specifically to one site in the genome. From the mapped transcripts, an average of 68% of the reads aligned to exonic regions, 29% to the intergenic regions and only 3% to intronic regions. The great percentage of transcripts mapped to intergenic domains is probably due to the incomplete genome annotation. Interestingly, commercial varieties Antequera and Bancal presented a significantly (*p* < 0.05) higher percentage (71.5%) of transcripts mapped to the exonic regions than the traditional ones Ardito and Magueija (61%). Concomitantly, the number of transcripts mapped to both intronic and intergenic regions is higher in the landraces. This may be explained by the fact that old traditional genotypes, collected in the 1930s, are more distinct from the reference genome than commercial varieties. Lastly, the results summarized in Figure 1A indicate that most reads (between 73 and 82%) were assigned as protein coding regions, the next most found class was non-translating–coding sequence (between 18 and 27%), and, in a very small amount ribosomal DNA (less than 3%).

Also a hierarchical clustering (Figure 1B) of sample-to-sample correlations revealed great intravarietal similarity between Antequera and Bancal samples, independently of the treatment, while for Ardito and Magueija, the similarities were greater between samples of the same condition (control/HT). In fact, the MDS (multi-dimensional scaling) plot grouping (Figure 1C) has shown that five of the six samples of each commercial varieties are closer to each other, while in landraces a clear separation between control and treatment samples was observed.

Differentially expressed genes (DEGs) between transcriptomes of immature grains from plants kept in control conditions and submitted to high temperature were considered significant with and adjusted *p*-value (padj) < 0.05 for all genotypes. Up and downregulated genes were obtained filtering the log2 foldchange absolute value higher than 1. For the four genotypes analyzed, a total of 10,366 DEGs were identified, 86% of them referent to Ardito and Magueija traditional genotypes, showing that they have a greater response to high temperature treatment. In a similar study done recently (Rangan et al., 2019), grain transcriptomes of three genotypes showed a higher number (more than 80%) of downregulated genes in susceptible genotypes, comparing with tolerant ones (2% of the DEGs were downregulated). Thus, the HT response of our landraces was similar to susceptible genotypes, as they present a higher number of DEGs.

**FIGURE 2:**
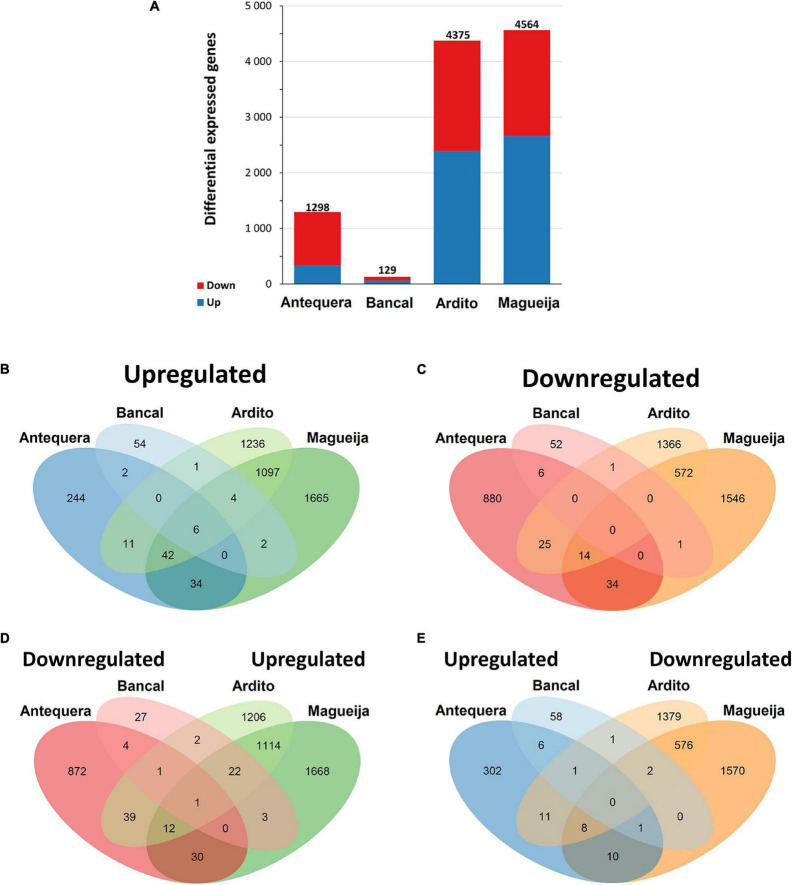
Differentially expressed genes (DEGs). **(A)** Number of DEGs between control and high temperature treated samples of commercial varieties Antequera and Bancal and landraces Ardito and Magueija. Red and blue indicate down and upregulated genes, respectively. **(B–E)** Venn diagrams of differentially expressed genes in commercial varieties Antequera and Bancal and landraces Ardito and Magueija: **(B)** upregulated in all genotypes; **(C)** downregulated in all genotypes; **(D)** downregulated in commercial varieties and upregulated in landraces; **(E)** upregulated in commercial varieties and downregulated in landraces. Non-overlapping regions represent the number of genes exclusive to one genotype. Overlapping regions indicate the number of genes common to two, three or four genotypes.

The number of DEGs is significantly different between all genotypes (*p* < 0.05), although these differences were less accentuated between Ardito and Magueija (Figure 2A). Particularizing to each genotype, the commercial variety Bancal presented the lower number of DEGs of all varieties studied, 129 in total, 69 upregulated and 60 downregulated. A ten times higher number of DEGs were identified in Antequera (1298), 339 upregulated and 959 downregulated. Considering the work above referred (Rangan et al., 2019), the higher number of DEGs detected in Antequera is in accordance with the previously reported worse heat response of this genotype in comparison to Bancal regarding grain protein content and grain yield (Tomás et al., 2020b). In Ardito, 4,375 DEGs were identified, 2,397 upregulated and 1,978 were downregulated. The genotype with greater number of DEGs (4,564) in response to high temperature treatment was Magueija, with 2,661 downregulated and 1,903 upregulated.

Our first approach was to investigate if any of A, B, and D genomes or distinct chromosomes were particularly affected by high temperature treatment, since is already documented that chromosomes 3A and 3B harbor genes involved in high temperature response [reviewed in Ni et al. (2018)]. Although, no significant differences were detected between genomes neither between chromosomes (Supplementary Figure 2).

The results presented in Figure 2B have shown that from the 1,199 upregulated genes common to more than one genotype, only six genes were common to Antequera, Bancal, Ardito, and Magueija. These six upregulated genes common to all genotypes (Table 1) encompassed annotated genes encoding three small heat shock proteins HSP20, one adenylate kinase, a BAG domain proteins and a ferritin. Adenylate kinase catalyzes a reversible transphosphorylation reaction that converts adenine nucleotides (ADP to ATP and AMP), and is critical for many processes in living cells (Pradet and Raymond, 1983), as, for example cellular activities maintenance during abiotic stress situations (Komatsu et al., 2014). BAG domain proteins are responsible for the modulation of chaperones activity as they bind to HSP70 proteins and promote the substrate release. Lastly, ferritin is a protein that functions in the iron storage in a soluble, non-toxic, readily available form. A recent study (Zang et al., 2017) showed that the overexpression of a gene encoding a ferritin (TaFER-5B) functionally complemented the heat stress-sensitive phenotype of a ferritin-lacking mutant of Arabidopsis enhancing heat, drought, oxidative, and excess iron stress tolerance associated with the ROS scavenging, as well as leaf iron content. Thus, the present work not only identified genes commonly modulated by HT in distant related hexaploidy wheats, but also pointed out an upregulated one that seem to be involved in HT response not only of wheat genotypes but also of dicot plants like Arabidopsis.

**TABLE 1:** Upregulated genes common to all genotypes analyzed.

Gene ID	Encoded protein
TraesCS3B02G155300	Adenylate kinase
TraesCS4D02G086200	Small heat shock protein (Hsp20 family)
TraesCS4A02G092900	Small heat shock protein (Hsp20 family)
TraesCS5A02G548000	BAG domain
TraesCS4B02G089800	Small heat shock protein (Hsp20 family)
TraesCS7D02G428200	Ferritin

There is a great difference between the number of upregulated genes common to both commercial varieties and the number of these genes common to both traditional ones, as can be seen in Figure 2B. Only 2 of the 418 (0.48%) HT upregulated genes were common in both commercial varieties. These genes encode for a protein induced by water deficit or abscisic acid stress and ripening, and a NAC transcription factor involved in plant development (NAC019-A1). A recent work revealed that this transcription factor is known to be a negative regulator of starch synthesis, kernel weight, and kernel width in wheat developing grains (Liu et al., 2020). In fact, our previous analyses of mature grains of these genotypes subjected to HT during grain filling revealed a reduction of starch amount in both commercial varieties and an increase in both landraces (Tomás et al., 2020a,b). On the other hand, a much higher proportion of upregulated genes, 1,097 of the 5,058 (21.7%) are shared by the landrace genotypes. These genes are associated with 1,747 biological processes gene ontologies, being the most represented terms related with protein folding and metabolic process.

Concerning downregulated genes, none was commonly detected in all genotypes, and it was also observed a much higher number of genes common to both traditional landraces (572–87.6%) than between commercial varieties (6–0.92%) (Figure 2C). These results reinforce the already referred suggestion that traditional genotypes have a more similar response to the HT treatment than commercial ones.

Among the 110 genes downregulated in commercial genotypes and upregulated in landraces (Figure 2D), there were genes encoding for several HSP of different classes, related with HT response proteins involved in nitrogen metabolism and seed storage proteins, that are mainly involved in the seed quality. On the other hand, only 34 genes were upregulated in commercial varieties and downregulated in traditional genotypes (Figure 2E), and the gene products are very diverse, encompassing proteins involved in DNA binding, zinc finger domains, transport proteins, and several No Apical Meristem (NAM) proteins, referred before as a negative regulator of starch synthesis (Liu et al., 2020).

Looking forward to unravel if there was any HT common response related with the more affected genes, we analyzed the ten most up and downregulated genes of each genotype (Supplementary Table 1). It was possible to note that in the commercial varieties, upregulated genes encode for diverse products, several involved in the RNA processing. For example, pentatricopeptide-repeat-containing proteins (PPR) were encoded by these upregulated genes in both commercial genotypes. They are known to influence the expression of several organellar genes by altering RNA sequence, turnover, processing, or translation (Barkan and Small, 2014). Also PPR proteins have crucial roles in response to different abiotic stresses in rice and were found as miRNAs target genes associated with thermotolerance in wheat (Tan et al., 2014; Chen G. et al., 2018; Ravichandran et al., 2019). On the other hand, 60% of landraces upregulated genes encode for products involved in heat shock response, as heat shock proteins or heat shock factors, well-documented as high temperature responsive genes [reviewed in Kaur et al. (2019)]. One of the Magueija up regulated genes is the already identified in leaves and roots TaHsfA6f, associated with increased thermotolerance (Xue et al., 2015; Bi et al., 2020) and, to our knowledge, it is for the first time identified in developing grains. As for the downregulated genes, the only characteristic that stood out was that in Antequera 7 out of the 10 downregulated genes encode for products related with protein synthesis and regulation, which can be related with the reduction in grain protein content observed in this variety after HT treatment (Tomás et al., 2020b).

### Functional Annotation and Gene Ontology Mapping of High Temperature Differentially Expressed Genes

**FIGURE 3:**
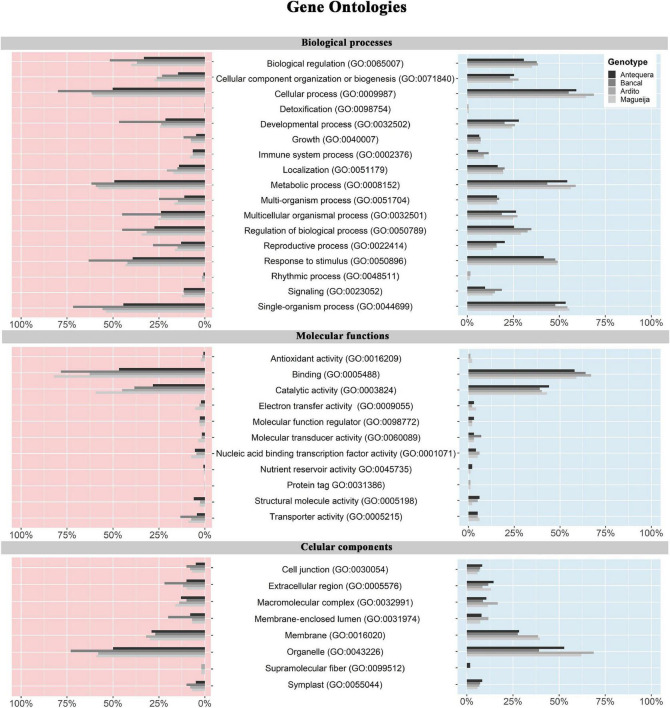
Gene ontology percentage of up and downregulated genes in commercial varieties Antequera and Bancal and landraces Ardito and Magueija assigned to second and third levels of biological processes, molecular functions and cellular component gene ontologies. Red and blue indicate down and upregulated genes, respectively.

In a more global perspective regarding each genotype response to high temperature treatment, functional annotation of DEGs of each genotype was made through the assignment of gene ontologies (GO) for biological processes, molecular functions and cellular components (Figure 3 and Supplementary Table 2). Figure 3 indicates the percentage of up and downregulated genes of each genotype, assigned to second and third levels of categories associated with each ontology. For all categories of the three ontologies the proportions of up and downregulated genes associated are very similar, being the classes with higher and lower number of genes the same in both cases for the four genotypes. This may indicate that, although the number of altered genes may be different in distinct genotypes (Figure 3), the functional roles in which the DEGs are involved constitute a common feature in wheat heat stress response.

In biological processes for both up and downregulated genes, the most represented categories are biological regulation (GO:0065007), cellular process (GO:0009987), metabolic process (GO:0008152), and response to stimulus (GO:0050896). It was also notorious that Bancal had a great percentage of downregulated genes assigned to several categories. For molecular functions ontology, catalytic activity and binding, mainly protein (GO:0005515) and organic cyclic compound binding (GO: 0097159) were clearly highlighted as compared with the other classes. Regarding cellular component GO, the most represented class was organelle (GO:0043226), with more than 50% of the DEGs in almost all the genotypes, and the other was membrane (GO:0016020) with half of this amount.

Several GO terms were significantly represented in each genotype (Supplementary Table 2), except for Bancal up regulated genes, that were only significantly enriched in 16 molecular function ontologies. In total 395, 129, and 154 distinct ontologies were identified for biological processes, molecular functions and cellular components, respectively. Ardito and Magueija present a closer response as several common ontologies were significatively represented, namely some categories of response to stress, establishment of cell polarity, protein complex biogenesis, *de novo* protein folding and carbohydrate catabolism for upregulated genes and DNA metabolism, regulation of gene expression and protein complex biogenesis for downregulated genes. We also found common categories in which Antequera and both landraces upregulated genes were enriched, such as protein folding, response to light and reactive oxygen species and heat acclimation biological processes and peroxisome and microbody cellular components. On the other hand, categories significantly enriched by downregulated genes common between commercial and landraces were only identified in cellular components, for instance related to nuclear lumen and thylakoid.

Particularly, from all the DEGs engaged in high temperature treatment response, 512 were assigned to response to heat category (GO:0009408), most of them presenting increased expression levels in the traditional varieties while in the commercial ones, only a small part was affected (Supplementary Table 3). These genes are related with other 106 biological processes, being the most represented protein folding (16%) and transcription regulation (8%), 41 cellular components with nucleus and integral component membrane being associated with a greater number of genes (14% each), and 105 molecular functions, being the most represented ATP binding (12.6%), protein binding (8.7%), and unfolded protein binding (7.3%). These classes of DEGs where also recently associated with wheat tolerance to drought during grain filling stage (Nouraei et al., 2022). Regarding unfolded protein binding, as expected, a great number of genes encode heat shock proteins (Hsp) and heat shock factors (Hsf). About 30% of the genes encode for the different Hsp20, Hsp40 (DNAJ domain), Hsp70 and Hsp90, and the great majority were upregulated in the traditional genotypes and remain unaltered or downregulated in the commercial ones. Also in the traditional genotypes, 12 genes encoding Apetala 2 proteins were identified as upregulated. Several proteins of this class were involved in grain and spike morphology, plant height, and spike emergence time determination, and play a key role in growth and development, including regulation of plant architecture and yield-related traits (Li et al., 2016; Zhao et al., 2019).

**FIGURE 4:**
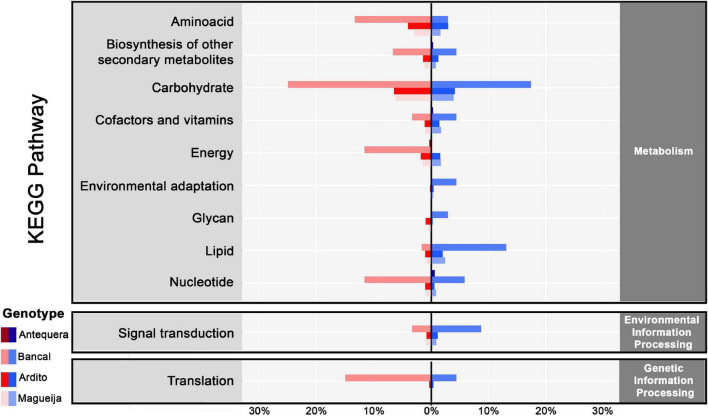
KEGG pathways enrichment percentage of up and downregulated genes in commercial varieties Antequera and Bancal and landraces Ardito and Magueija associated with metabolic, environmental and genetic information processing pathways. Red and blue indicate down and upregulated genes, respectively.

To further disclose biological functions of DEGs and determine if any pathway have a significant involvement in heat tolerance, we investigated DEGs involved in Kyoto Encyclopedia of genes and Genomes (KEGG) pathways and 749 DEGs were assigned related with 107 KEGG pathways (Figure 4 and Supplementary Table 4). Antequera was the genotype with less DEGs associated with these pathways (0.5%), the traditional genotypes revealed 9% each, and Bancal was the genotype that presented the higher percentage (57%).

Analyzing the pathways associated with products encoded by downregulated genes, the ones related with carbohydrate metabolism were the most influenced in Bancal and both traditional genotypes. The only carbohydrate pathway associated with downregulated genes of all four genotypes were the Glycolysis/Gluconeogenesis pathway, although neither the genes nor the encoded enzymes were common. Although, inside this category, the majority of Bancal downregulated genes encoded for enzymes involved in pentose and glucuronate interconversions pathway and in landraces encoded for starch and sucrose metabolism, with the majority of encoded enzymes associated with glucose synthesis. Some of the enzymes categorized in the pentose and glucuronate interconversions were pectinesterases known to be involved in cell wall remodeling that occurs during high temperature response (Wu et al., 2018). Kino et al. (2020) reported also a downregulation of genes involved in pericarp cell wall expansion due to high temperatures exposure during post anthesis, and speculate that this can be related with the reduction in grain weight observed after this stress. Our work also corroborated this suggestion since the majority of DEGs encoding pectinesterases were downregulated in landraces in which a reduction in grain weight was observed (Tomás et al., 2020a). The second most affected pathways were the ones involved in amino acid metabolism, with the majority of DEGs assigned to cysteine and methionine metabolic pathways for Bancal and both landraces. This was an unexpected result as the accumulation of this amino acid was reported in high temperature conditions (Tao et al., 2018). Again, only one pathway, the glycine, serine and threonine metabolism, was identified as being associated with downregulated genes in all the genotypes, but also again none of these genes was common to all the genotypes. An interesting result was the percentage of Bancal downregulated genes encoding for Aminoacyl-tRNA synthetases (nine different genes encode for six different synthases), classified in the translation pathways. This was not an expected result as several works in distinct species report an increase in different enzymes of this family in abiotic stress situations (Giritch et al., 1997; Thimm et al., 2001; Kobayashi et al., 2005; Baranašić et al., 2021). Lastly, several downregulated genes in Bancal were associated with nucleotide metabolism, more specifically with purine metabolism, and encoded for Adenosine triphosphatase (ATPase).

Upregulated genes were also associated with most of the mentioned pathways for downregulated genes. In fact, the encoded products were in some cases the same as for downregulated genes, suggesting that they include several cases of different enzyme isoforms or homologous genes with different functions, as already reported (Liu et al., 2015; Kaushik et al., 2020). Carbohydrate pathways include the greater number of associated upregulated genes for Bancal and both landraces. Particularizing, starch and sugar pathway was the most common, and glycolysis was the second. For Bancal, nucleotide metabolism was again the pathway with the higher percentage, although the encoded enzymes were involved in the dephosphorylation of ATP molecules, as well as translation pathways, in which were detected transcripts for enzymes involved in glutamate and tryptophan tRNA synthesis. Upregulated genes of Bancal and both landraces encoded also for enzymes in lipid metabolism. Specifically involved in cutin, suberine and wax biosynthesis, glycerolipid metabolism, fatty acid elongation, fatty acid biosynthesis, the latter two being related only with upregulated genes in landraces. This may indicate an alteration in lipids proportions in response to high temperature as previously reported [reviewed in Abdelrahman et al. (2020)].

### High Temperature Effects in Storage Proteins Encoding Genes

**FIGURE 5:**
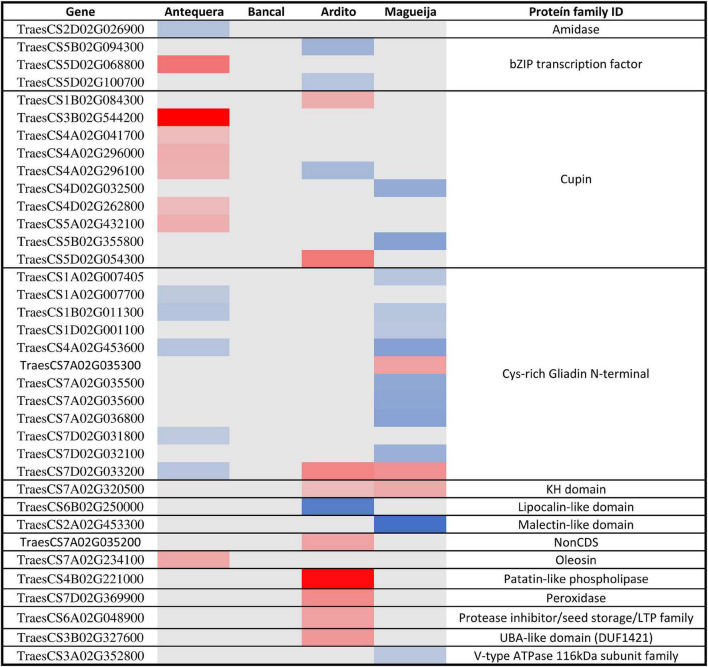
Differentially expressed genes involved in nutrient reservoir activity ontology in commercial varieties Antequera and Bancal and landraces Ardito and Magueija. Red and blue indicate down and upregulated genes, respectively, and color intensity are related with the degree of gene expression alteration; gray represents unaltered genes.

Gluten is determinant for the wheat suitability to produce bread as it is a protein network that entrains air bubbles during dough fermentation. It is composed from two classes of storage proteins, glutenins, responsible for the dough strength and elasticity, and gliadins which confer extensibility and viscous properties to gluten required for dough development. Gliadin/glutenin ratio is determinant for rheological characteristics (Dhaka and Khatkar, 2015), being for that reason important to access if these proteins encoding genes’ are affected by high temperature treatment. Storage proteins encoding genes are classified in the nutrient reservoir activity ontology and the expression levels of DEGs associated with this category are presented as heatmap in Figure 5. None of the genes presented altered expression in Bancal genotype. Additionally, about 60% of the DEGs were related with two protein families, Cupins and Gliadins.

The results obtained revealed 12 gliadin encoding genes differentially expressed, mostly upregulated in Magueija and Antequera, that may have implications in grain quality. In fact, several studies showed that an increase in gliadin fraction has a detrimental effect on the technological characteristics of wheat. Flours with higher gliadin content present weaker gluten quality and dough, with increased viscosity and stickiness (Barak et al., 2015). Additionally, Antequera and Ardito presented 6 and 2 downregulated genes encoding for Cupins, respectively, while three genes were upregulated, 1 in Ardito and 2 in Magueija. Cupins were already described as heat responsive proteins with an unusual thermostable character which facilitates their accumulation in a number of heat-stressed organisms (Dunwell et al., 2001). A more recent work shows that these proteins are preferentially accumulated when protein synthesis components are generally decreased during heat stress, suggesting that they may provide valuable insights into improving the protein content of wheat (Wang et al., 2018). A significative reduction of protein content was previously observed in Antequera mature grains after heat stress treatment (Tomás et al., 2020b). Altogether, these results show that gliadins are more affected by high temperature treatment than glutenins in both wheat commercial varieties and landraces and reinforce the need to investigate the cupins role in heat stress response.

It must be emphasized that the expression levels of genes related with flour quality, like the ones encoding glutenins and enzymes involved in starch and puroindolines synthesis, were already evaluated through qRT-PCR (Tomás et al., 2020c), and the results obtained confirm the RNA Sequencing analysis here presented.

**FIGURE 6:**
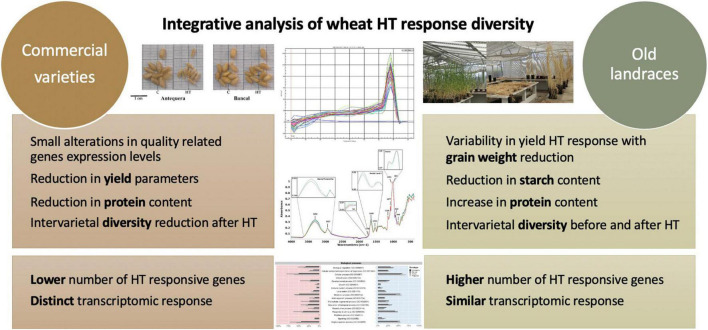
Integrative analysis of grain yield, composition and transcriptomic analysis of the commercial varieties and the old landrace responses to heatwave at the grain-filling stage.

Overall, the results obtained in this and past works (Tomás et al., 2020a,b,c) show that high temperature treatment tend to reduce yield and quality differences observed between commercial varieties in control conditions. Conversely, differences observed between old landraces were enhanced in plants submitted to HT treatments (Figure 6). Characteristics like grain weight, protein content and transcription profiles of heat responsive genes on the traditional genotypes studied encourages a deeper analysis of those genotypes enclosed in Vasconcellos collection (Vasconcellos, 1933). Nevertheless, accordingly to all our analysis, the commercial variety Bancal seems to be a promising genotype to cope with high temperatures.

Several questions arise from this work, confirming that high temperature response results from a complex of physiological, cellular and molecular processes, as previously proposed (Jacott and Boden, 2020; Schaarschmidt et al., 2021). Though, several pieces are missing to compose the intricate puzzle of plant response to this abiotic stress (Jagadish et al., 2021; Khan et al., 2021). A deeper exploitation of RNA sequencing data, focusing on particular pathways, will be needed to enrich correlations between specific changes in genes expression profiles and phenotypic alterations induced by heat wave like treatments.

## Data Availability Statement

The original contributions presented in this study are publicly available and sequences data can be found here: https://www.ncbi.nlm.nih.gov/search/all/?term=PRJNA750265.

## Author Contributions

DT and MS: conceptualization, methodology, and validation. DT: formal analysis, investigation, visualization, and writing—original draft preparation. MS: funding acquisition and project administration. MS and WV: supervision and writing—review and editing. All authors contributed to the article and approved the submitted version.

## Conflict of Interest

The authors declare that the research was conducted in the absence of any commercial or financial relationships that could be construed as a potential conflict of interest.

## Publisher’s Note

All claims expressed in this article are solely those of the authors and do not necessarily represent those of their affiliated organizations, or those of the publisher, the editors and the reviewers. Any product that may be evaluated in this article, or claim that may be made by its manufacturer, is not guaranteed or endorsed by the publisher.

## Footnotes

1
ftp://ftp.ensemblgenomes.org/pub/plants/release-48/fasta/triticum_aestivum/dna/Triticum_aestivum.IWGSC.dna.toplevel.fa.gz

2
http://systemsbiology.cau.edu.cn/agriGOv2/index.php

3
http://geneontology.org

4
http://www.kegg.jp/kegg
