# Supplementary material for: Grain Transcriptome Dynamics Induced by Heat in Commercial and Traditional Bread Wheat Genotypes
Source: Front Plant Sci. 2022 Jun 17;13:842599. doi: 10.3389/fpls.2022.842599 (PMC9248373; doi:10.3389/fpls.2022.842599)
Supplement: Supplementary file 6 [file Image_1.pdf]

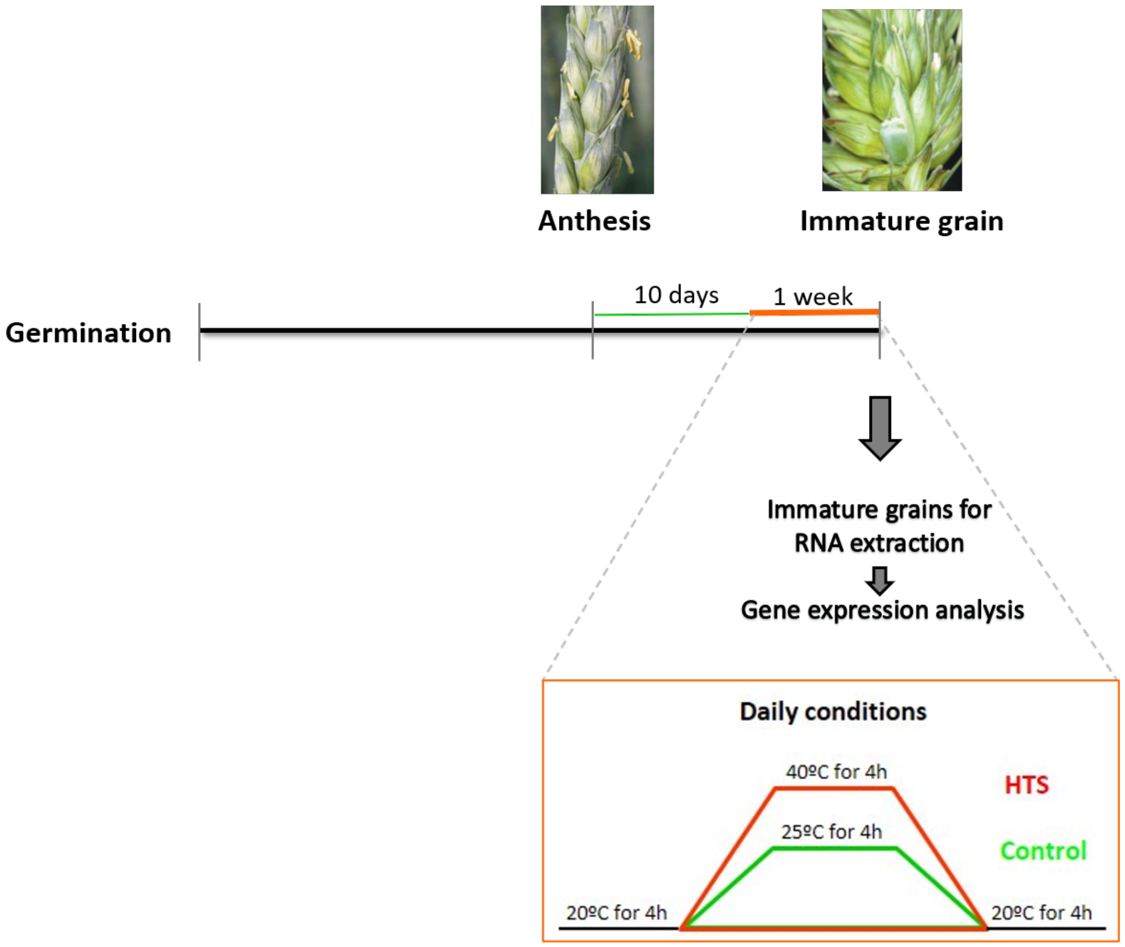

Supplementary Fig. S1 - High temperature assay scheme. Representation of assay conditions and immature grains collection timepoint for further RNA extraction (adapted from Tomás et al., 2020a).
