# Supplementary material for: Grain Transcriptome Dynamics Induced by Heat in Commercial and Traditional Bread Wheat Genotypes
Source: Front Plant Sci. 2022 Jun 17;13:842599. doi: 10.3389/fpls.2022.842599 (PMC9248373; doi:10.3389/fpls.2022.842599)
Supplement: Supplementary file 7 [file Image_2.pdf]

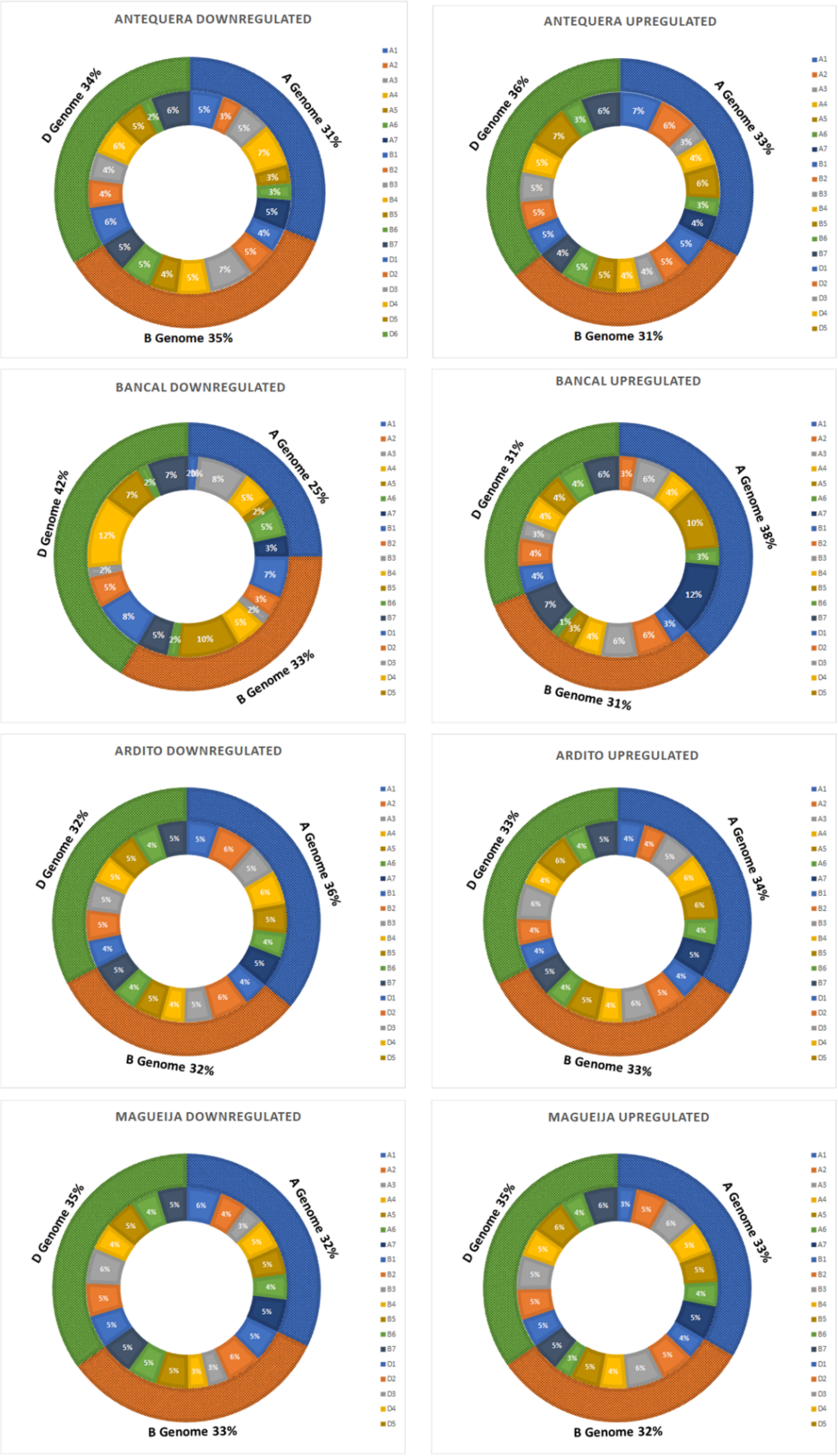

Supplementary Fig. S2 – Genome/chromosome locations of differentially expressed genes. Schematic representation of the genomic position of DEG on all chromosomes of commercial varieties Antequera and Bancal and landraces Ardito and Magueija. The three genomes (A, B and D) are displayed in the outer circle, and the chromosomes 1 to 7 of each genome are displayed in the inner circle. In both cases the percentage of associated DEGs is displayed.
